# Medical and Philosophical Intelligence

**Published:** 1818-10

**Authors:** 


### MEDICAL AND PHILOSOPHICAL INTELLIGENCE.

AN article in the last number of the Medical Repository, by Mr. Diamond, contains some remarks on a passage in the paper of Mr. Hey, *on the effects of the Venereal Disease on the Fœtus in Utero*; (published in the seventh volume of the Medico-Chirurgical Transactions;) which we should hardly have expected to have witnessed at the present period. Mr. Hey observes, "It may justly excite surprise, that the gentleman whose case I have just related, should have remained free from disease when his wife was in the condition which I have described:" (i. e. in the opinion of Mr. Hey, affected with syphilis). On this Mr. Diamond remarks, "I ought to observe here, for the information of those who have not seen the case published by Mr. Hey, that the infection was communicated by the gentleman to his wife, although he had no symptoms of the disease on him at the time of his marriage. I believe I have myself seen cases of the same nature." Our readers will doubtless be surprised, that a person unacquainted with the circumstances of the case from actual observation, could make so dogmatical an assertion; it is improper, even were the opinion advanced founded on observation, but when there are so many other obvious ways of accounting for the circumstances stated in the paper in question, such an assertion must be considered still more extraordinary. It would have been more judicious to have endeavoured to ascertain the real nature of the disease. We notice this paper because of the following question, proposed by the author;—"In what manner is the venereal disease communicated to a female by a male, when he shall at the same time have no symptom of the complaint on him. *That such cases do occur is beyond the possibility of a doubt!!* and it would afford me much satisfaction to see the subject discussed in the Medical Repository." Such questions, we fear, will be proposed, and such bold unfounded assertions made; while every eruptive disease that is not understood is called syphilis, and while indolence and vanity, instead of a disposition for the patient investigation of facts and an inclination to receive instruction from others, influence the conduct of practitioners of surgery. Were the works of Mr. Hunter, and the facts he has established, as well understood as they are promptly quoted, such a question would not have appeared to disgrace the medical science of the present day.

The same Journal contains a case, related by Mr. Sterndale, jun. Sheffield, as *illustrating the Hereditary Nature of Bronchocele.*

The writer first alludes to an instance related by Dr. Brischet, of Paris, of an infant being born with the thyroid gland unusually large, whose mother was affected with bronchocele. Mr. Sterndale then states that he had been requested to see a child about ten days after its birth, which was born with what the nurse called a *Derbyshire* neck; this was enlargement of the thyroid gland: the mother was labouring under bronchocele in a very advanced state.

We do not consider that the above cases demonstrate the transmission of *disease* from the mother to the offspring; but only that similarity of external form of body, which is so often witnessed both in the human race and brute animals. It is well known to breeders of cattle, that, if an animal from accident have any peculiarity of form,—for instance, if a sheep be permanently deprived of a horn, that the offspring will frequently evince the same deformity. We know a woman, who, having lost the fore-finger of the left hand, produced four children with the same deprivation; while two, born intermediately with the others, were properly formed. Instances of increase of bulk, or form of parts, being similarly transmitted, cannot indeed be produced, very probably, because such an alteration of parts is of rare occurrence. We think that it yet remains to be proved, by observation, whether or not such an enlargement in the thyroid gland of an infant newly born be actually *disease*.

A case is inserted in the above Journal, by Mr. Taunton, which he considered to be elephantiasis. The patient was a female, aged fifty-five; the disease came on after sitting-up a week with a person who was ill: the leg became cold and numbed, and nearly useless; it soon after began to swell, and, in two or three years, had become considerably increased in size; the skin was rough, thick, scaly, and irregularly of a dark brown colour. The woman died eleven years after the appearance of the disease. The limb, after death, was nineteen inches and a half in circumference below the ankle, above it twenty-one and a half, and about the middle of the leg twenty-two inches. There was no disease of any of the viscera that appeared to be connected with that of the limb.

---

One of three persons bitten by a rabid dog, at Knightsbridge, on the 17th of September, did not discover, till four days after, that his prepuce had been torn by the teeth of the animal. He had wounds in his leg, for which excision of the parts had been effected almost immediately after the receipt of the injury; the same practice was adopted with the other patients. The result of this case may contribute something to our knowledge respecting the efficacy of excision of the injured parts. The dog was killed and dissected: the principal morbid appearances were marks of previous inflammation of the stomach.

---

A physician of considerable talents, and whose opportunities for observation are ample, has directed a share of his attention to the effects of the antimonial powder of the London Pharmacopœia: the result of his enquiries has led him to believe, that it is as generally obtained almost as an inert preparation. The tartarized antimony, he considers, is a more efficacious medicine, and more certain in its operation.

The use of tartarized antimony has lately been carried to a great extent in public practice in London. A grain has been administered every one or two hours in continued fever, without producing vomiting after the first or second dose. The Italian physicians place great reliance on this medicine, and not unfrequently direct a grain to be given every hour. Our own observation has led us to estimate this remedy very highly, when administered in the mode above mentioned. It diminishes the powers of life in a remarkable degree, and particularly when combined with digitalis; its effects should, therefore, be cautiously watched when given to such an extent.

---

The following proposal for a new *mode of preserving vegetable remedies*, has been made by Dr. Marshall Hall.

"It is an object of much regret," he observes, "that all the modes of preparing vegetable remedies hitherto employed are defective; and that no mode of preserving these substances, with their virtues unimpaired, should have been discovered. Sometimes the process of preparation injures the virtues of the remedy, or extracts them partially only; in other cases, their subsequent preservation is imperfect. Dried vegetable remedies, extracts, tinctures, infusions, and decoctions, are all liable to one or more of these objections. Might not some of them be preserved without subjecting them to any previous process, or to the action of any external agent, by which their virtues are partially destroyed, or only partially extracted? In the case of digitalis, cicuta, hyoscyamus, &c. the writer has taken the fresh herb, collected free from dew, and having rubbed the leaves into the finest pulp, he has simply formed a properly consistent mass, by the addition and careful intermixture of white sugar, or of dried soap. In this manner the vegetable may be long preserved; and the advantages are obtained of administering it throughout the year in its pristine state, and without previously subjecting it to any operation, or to the agency of any substance by which its properties might be enfeebled or destroyed. It must remain to be decided which of the two modes is the preferable one, and whether each may not be better adapted than the other for the preservation of particular substances. Either compound may be formed into pills, or mechanically suspended in a draught or mixture."

---

Some chemical physicians of London have lately instituted a series of experiments with the pyro-ligneous acid, to ascertain its power of correcting and preventing the decomposition of animal substances. Meat, almost in a state of putrefaction, on being washed over with it two or three times, was rendered perfectly sweet and palatable. The flavour it gave to some salted tongues, that were previously unfit for use, was particularly agreeable, somewhat similar to that produced by smoaking them. When meat contains maggots, or the larvæ of insects, it will be necessary to wash it over first with muriatic or nitric acid, to destroy them, which the pyro-ligneous acid will not effect.

It seems probable, that the use of this acid, for the preservation of meat for sea-voyages, might be adopted with advantage. It may be obtained for somewhat less than one shilling a gallon; and is not unfrequently sold, diluted, as distilled vinegar.

Some very interesting remarks on the vegetable productions discovered in the neighbourhood of the River Zaire, in Africa, are made by Mr. Brown, in his essay published in Captain Tuckey's narrative; the most important of which we shall transcribe for such of our readers as feel pleasure in contemplating this part of the organic productions of nature.

From the River Senegal in 16° north latitude, to the Zaire in 6° south latitude, there is a remarkable uniformity in the vegetation, not only with respect to the natural orders and genera, but even as far as respects the species. The same similarity seems also to exist in the cultivated as well as in the indigenous plants. On the banks of the river, as far as the expedition proceeded, the principal articles of vegetable food were found to be Indian corn, cassava, two kinds of pulse, and nuts. The most valuable fruits were plantains, papas, pumpkins, limes, oranges, pine-apples, tamarinds, and a fruit called safa, like a small plum. A very valuable plant along the whole line of coast is the oil-palm (*elæis guineensis*,) from which the best kind of palm wine is produced. It appears also that the common yam, capsicum, two species of the sugar-cane, and tobacco, were cultivated in particular spots. It is worthy of remark, that the greatest part of these cultivated plants are not natives of the country, but have been introduced from other parts of the world; maize, cassava, pine-apple, papaw, capsicum, and tobacco, were probably introduced from America; while the banana, lime, orange, tamarind, and sugar-cane, are probably of Asiatic origin. In determining to what country we are to refer the origin of plants that are now dispersed in various situations, Mr. Brown remarks, that we may be assisted by a careful examination of the geographical distribution of genera. We may, at least, go so far as to conclude, that, in doubtful cases, it is more probable that the plant in question should belong to that country in which all the other species of the same genus are found decidedly indigenous, than to that where it is the only species of the genus known to exist. Exceptions to this general rule will probably be found; the cocoa-nut, for example, there is every reason to believe is indigenous in the islands of equinoctial Asia, yet it is the only species of its genus which does not exclusively belong to America. There are some valuable plants that are cultivated in most of the western parts of Africa, yet were not observed in Congo; of these, the most remarkable are the cocoa-nut, rice, and the sweet potato (*convolvulus batatas*). We have no means of determining the relation which the vegetation of the eastern shores of Equinoctial Africa bear to the western. The flora of Abyssinia, as ascertained by Mr. Salt, has not much connexion with that of Congo, and the flora of Egypt has still less. The plants of Congo appear likewise to be very different from those of the south of Africa, containing none of the more remarkable genera and orders that are found in this district: there seems also to be very little affinity between the vegetables of Congo and those of Madagascar, and the islands of France and Bourbon, and considerately more with those of India; while, on the contrary, they have less affinity with those of equinoctial America. It is observed, however, that there are several genera common to this part of Africa and America that have not yet been observed in India or New Holland; and there are more than thirty species in Professor Smith's collection, which are also natives of the opposite coasts of Brazil and Guiana. It has been questioned whether there be any species, especially of the dicotyledonous plants, which are found in the equatorial districts both of the old and new continent. In order to elucidate this point, Mr. Brown has formed a list of the plants in Professor Smith's collection, which are common to Africa, America, and Asia; afterwards, a list of those that are common to Africa and America, but are not found in India; and a third list of those that are indigenous in Africa and India, but not in America. The greater part of the species enumerated in these lists are strictly equinoctial plants, although few of them have been found in the southern parts of the temperate zone. Mr. Brown remarks, that some or the whole of these plants may be conceived to have been originally natives of only one of these countries, and may have been conveyed to the other either by accident or design; but he offers many considerations which render it probable, that, with respect to the greatest part of them, this is not the case.

The Surrey Dispensary is now open to the admission of physicians' pupils, who are entitled to attend the medical practice of the institution.

We regret to announce the death of Samuel Merriman, M.D. who died at his house in Half-moon Street, aged 87. This gentleman was the author of several Essays on medical subjects, and was highly respected by the profession.—Also, in Southampton-row, 90, J. Wilkinson, M.D. F.R.S. and A.S. author of some poems.

### REPORT OF DISEASES.

THE prevalence of continued fever has diminished during the last month, and these cases have generally terminated more favourably than those of the period embraced by our last Report: symptoms of local inflammation have been more strongly evinced, and the nervous system has appeared less disturbed. The organs principally affected were, the lungs and brain. Inflammation of the mucous membrane of the intestines has rarely occurred, except in cases of relapse: these cases have terminated favourably under the use of an emetic followed by purgatives, leeches to the abdomen, and cold affusion. This state of disease should be cautiously investigated, or the absence of pain, except on pressure being made on the parts, may mislead the medical attendant. The respiratory organs were frequently the seat of inflammation, in relapses during the early part of the month; and, from this occurring before the strength of the patient had become re-established, much caution was required in the treatment. The favourable results we have witnessed in these cases from the use of tartarized antimony in large and frequent doses, a single copious bleeding being generally premised, make us particularly desirous to direct the attention of our readers to this powerful remedy. In a severe case of laryngitis affecting a young female, occurring about ten days after convalescence from continued fever, it was given to the extent of twelve grains between the hour of eight in the evening and noon of the following day, when all the alarming symptoms had disappeared; (she had previously lost twenty-five ounces of blood, which produced only temporary relief;) the voice still remained affected, but respiration and deglutition were performed without much difficulty: the pulse was reduced to sixty in a minute. No vomiting had occurred during the last eight hours; in two days more, she was convalescent. In the exhibition of this remedy, the periods between each dose should not exceed two hours, otherwise vomiting will be repeatedly excited; the dose for an adult may be one grain.

Scarlatina and measles, in a mild form, have occasionally appeared, but not so frequently as to be considered among the prevalent diseases.

Hæmoptysis has been of rather frequent occurrence; a considerable proportion of the cases were observed in middle-aged persons who had been subject to periodical evacuations from hæmorrhois, which had ceased during the summer: these would probably have been prevented by the old salutary practice of taking physic at the commencement of spring and autumn.

Acute rheumatism is more prevalent than usual at this period of the year.

Diarrhœa has been common, and, in many cases, very severe; in a few instances, it has terminated fatally, exhausting the vital powers with extraordinary rapidity.

Many anomalous cutaneous diseases, some bearing a resemblance to *Lichen*; others, termed syphilitic by routine practitioners; have lately appeared: the warm bath, purgatives, and small doses of tartarized antimony have rarely failed to relieve them.

### METEOROLOGICAL JOURNAL,

By Messrs. WILLIAM HARRIS and Co. 50, Holborn, London.

*From the 20th August to the 19th September, inclusive.*

| Day of Month. | Moon. | Rain gauge | THERM. | | | BAROM. | | De Luc's Hygrom. Dry | | Damp | | Wind. | | Atmospheric Variation. | | |
|---|---|---|---|---|---|---|---|---|---|---|---|---|---|---|---|---|
| Aug. | | | | | | | | | | | | | | | | |
| 20 | | | 55 | 61 | 48 | 29·95 | 29·93 | | | 5 | 3 | NE | N | Fine | | |
| 21 | | | 57 | 62 | 49 | 29·89 | 29·96 | | | 4 | 5 | NE | NNW | Fine | | |
| 22 | | | 55 | 63 | 53 | 30·00 | 30·03 | | | 9 | 9 | NE | NE | Fine | | |
| 23 | ○ | | 60 | 67 | 51 | 30·03 | 30·09 | | | 11 | 9 | N | N | Fine | | |
| 24 | | | 60 | 69 | 58 | 30·15 | 30·08 | | | 7 | 5 | NNW | SW | Clo. | Fine | |
| 25 | | | 62 | 68 | 59 | 30·07 | 30·05 | | | 3 | 5 | W | NNW | Clo. | Fine | Clo. |
| 26 | | | 65 | 70 | 60 | 30·00 | 29·92 | | | 7 | 9 | W | W | Clo. | Fine | Clo. |
| 27 | | | 63 | 71 | 60 | 29·85 | 29·75 | | | 9 | 5 | W | SW | Clo. | Rain | Clo. |
| 28 | | | 65 | 70 | 61 | 29·71 | 29·84 | | | 3 | 5 | W | W | Fine | | |
| 29 | | | 65 | 72 | 58 | 29·90 | 29·95 | | | 7 | 5 | W | SW | Fine | | |
| 30 | | | 61 | 68 | 61 | 29·96 | 29·98 | | | 3 | 3 | NW | WNW | Rain | Fine | |
| 31 | ● | ,02 | 63 | 70 | 57 | 29·94 | 29·85 | | | 7 | 5 | SE | E | Fine | | |
| Sept. | | | | | | | | | | | | | | | | |
| 1 | | ,01 | 61 | 68 | 58 | 29·58 | 29·70 | | | 7 | 9 | SW | S | Rain | Clo. | Rain |
| 2 | | ,08 | 62 | 70 | 60 | 29·87 | 29·95 | | | 9 | 7 | W | SW | Fine | | |
| 3 | | | 63 | 72 | 60 | 30·00 | 30·01 | | | 9 | 5 | SE | S | Fine | | |
| 4 | | | 63 | 70 | 62 | 30·00 | 29·91 | | | 10 | 19 | SE | S | Clo. | Fine | |
| 5 | | | 65 | 70 | 60 | 29·87 | 29·82 | | | 20 | 17 | S | SW | Rain | Clo. | Rain |
| 6 | | ,09 | 63 | 71 | 55 | 29·72 | 29·78 | | | 20 | 25 | W | WSW | Rain | Fine | |
| 7 | ○ | ,05 | 58 | 67 | 53 | 29·90 | 29·92 | | | 15 | 9 | W | W | Fine | | Clo. |
| 8 | | | 58 | 66 | 53 | 29·90 | 29·81 | | | 10 | 12 | W | NE | Fine | Rain | Clo. |
| 9 | | ,10 | 58 | 61 | 48 | 29·71 | 29·65 | | | 15 | 16 | NE | NNE | Fog | Rain | Clo. |
| 10 | | ,07 | 52 | 57 | 48 | 29·78 | 20·83 | | | 15 | 16 | W | NW | Fine | | |
| 11 | | | 52 | 60 | 48 | 29·87 | 30·93 | | | 10 | 10 | WNW | W | Fine | | |
| 12 | | | 53 | 64 | 51 | 30 00 | 30·05 | | | 10 | 12 | W | WNW | Fine | | Clo. |
| 13 | | | 58 | 67 | 51 | 30·15 | 30·27 | | | 13 | 12 | N | NNE | Fine | | |
| 14 | ○ | | 57 | 65 | 50 | 30·21 | 30·07 | | | 11 | 10 | SW | SW | Fog | Fine | Clo. |
| 15 | | | 54 | 59 | 48 | 29·78 | 29·76 | | | 15 | 10 | SW | SSE | Clo. | | |
| 16 | | | 49 | 61 | 49 | 29·62 | 29·39 | | | 12 | 15 | SSW | SW | Fog | Clo. | Finc |
| 17 | | .19 | 55 | 60 | 48 | 30·19 | 30·24 | | | 17 | 20 | S | S | Rain | Fine | Clo. |
| 18 | | ,23 | 53 | 59 | 49 | 30·10 | 30·00 | | | 16 | 12 | E | SE | Rain | | Clo. |
| 19 | | ,31 | 56 | 62 | 48 | 30·05 | 30·00 | | | 19 | 25 | S | SE | Rain | Clo. | Rain |

The quantity of rain fallen in the month of August is 4-100ths of an inch only.

